# The Language of Change Among Individuals With Mild Intellectual Disability or Borderline Intellectual Functioning: Client Responses to Therapist Motivational Interviewing Skills

**DOI:** 10.1111/jar.70169

**Published:** 2026-01-10

**Authors:** Lotte C. F. Gosens, Jannet M. de Jonge, Robert Didden, Roy Otten, Evelien A. P. Poelen

**Affiliations:** ^1^ Research & Development Pluryn Nijmegen the Netherlands; ^2^ Behavioural Science Institute, Radboud University Nijmegen the Netherlands; ^3^ ACHIEVE, Centre of Applied Research, Faculty of Health, Amsterdam University of Applied Sciences Amsterdam the Netherlands; ^4^ Trajectum Zwolle the Netherlands

**Keywords:** change talk, mild intellectual disabilities, motivational interviewing, substance use disorder

## Abstract

**Background:**

The link between a therapist's motivational interviewing skills and the subsequent response of individuals with mild intellectual disabilities to borderline intellectual functioning (MID‐BIF) is unknown. This study examines this sequential relationship and describes change talk (CT) in individuals with MID‐BIF during substance use disorder (SUD) treatment.

**Method:**

In this study, 35 treatment sessions were sequentially coded using the Motivational Interviewing Sequential Code for Observing Process Exchanges. Observed and expected frequencies of transitions, transitional probabilities, odds ratios and 95% confidence intervals were calculated in Generalized Sequential Querier software. Furthermore, frequencies of CT in IBM SPSS statistics were calculated.

**Results:**

Questions and reflections of CT and two‐sided questions were followed by CT. Questions and reflections of counter change talk (CCT) and two‐sided questions were followed by CCT. Individuals expressed all kinds of CT utterances.

**Conclusions:**

Questions and reflections of CT are powerful skills in evoking CT in individuals with MID‐BIF during SUD treatment.

## Lay Summary

- The present study contributes to the understanding of MI and CT in individuals with MID‐BIF and SUD.
- Questions of CT and reflections of CT are powerful skills in evoking CT in individuals with MID‐BIF during SUD treatment.
- Individuals with MID‐BIF are able to express CT with their own words and sometimes need the therapist to provide words for their desire.

## 1 Introduction

Prevalence rates of substance use disorder (SUD) in individuals with mild intellectual disability or borderline intellectual functioning (MID‐BIF; IQ range: 50–85) are high (van Duijvenbode and VanDerNagel 2019). In addition, in addiction care approximately 30%–40% of the individuals have a MID‐BIF (Didden et al. 2020; van Duijvenbode and VanDerNagel 2019; van Duijvenbode et al. 2015). Motivational interviewing (MI) is widely used in SUD treatment, and many studies have shown its effectiveness in decreasing substance use (SU) (Riper et al. 2014; Smedslund et al. 2011). The scarce number of studies that have evaluated the effectiveness of MI in individuals with MID‐BIF showed that MI is feasible (Kouimtsidis et al. 2017), increases motivation and changes motivation to more autonomous motivation (Frielink et al. 2015; Mendel and Hipkins 2002), and is promising in decreasing SU in combination with cognitive behavioural therapy (CBT) (Gosens et al. 2024).

MI is a client‐centred approach to strengthen a client's motivation and commitment to change behaviour (Miller and Rollnick 2023). By using MI, the therapist explores the client's reasons to change, detects ambivalence, evokes change talk (CT) and reduces counter change talk (CCT). CT comprises all the client's language that expresses statements about change (Martin et al. 2005; Miller and Rollnick 2023). In contrast, CCT comprises all the client's language that expresses statements counter to change (Martin et al. 2005; Miller and Rollnick 2023). An MI therapist uses the MI skills within an MI attitude. MI prescribes MI‐consistent skills, such as affirming, emphasising control, seeking permission, supporting, advising with permission, questioning and reflecting. MI‐inconsistent skills, such as advising without permission, confronting, directing, giving opinions and warning are not preferable. The attitude in MI is named the ‘MI‐spirit’, consisting of partnership (i.e., an equal relationship between client and therapist, using the expertise of both), acceptance (i.e., non‐judgement), compassion (i.e., commitment to the best interest of the client) and empowerment (i.e., strengthening the client's strengths and abilities) (Miller and Rollnick 2023). In MI, the therapist completes four tasks: engaging (i.e., trusting relationship), focusing (i.e., treatment goal), evoking (i.e., motives and commitment for treatment goal) and planning (i.e., establishing change plan) (Miller and Rollnick 2023).

Meta‐analyses have shown evidence for the causal relationship in which MI therapist skills are associated with client language, and client language is associated with behaviour change (Magill et al. 2018, 2014). Several studies investigated sequential associations between therapist language and client language. Specifically, they showed that MI‐consistent therapist statements were followed by CT (Gaume et al. 2008). Moreover, therapists' reflections of CT and questions of CT were followed by CT, while therapists' reflections and questions of CCT were followed by CCT (Barnett et al. 2014; Berman et al. 2019; Lindqvist et al. 2017). Research testing the association between client language and SU outcome showed associations between CT and a decrease of SU at follow‐up (Houck et al. 2018; Vader et al. 2010). In contrast, CCT was related to increased SU during treatment (Houck et al. 2018) and at follow‐up (Vader et al. 2010). In addition, more MI‐inconsistent language by therapists was associated with increased SU at 2‐month follow‐up (Rodriguez et al. 2017).

When adapted, MI has been shown to be feasible in individuals with MID‐BIF (Frielink et al. 2015; Gosens et al. 2024; Kouimtsidis et al. 2017; Mendel and Hipkins 2002). However, studies testing associations between therapist language and client language during MI in individuals with MID‐BIF are lacking. Individuals with MID‐BIF may differ from individuals without MID‐BIF in client language. A common challenge in MI for the therapist is to recognise even small CT or disguised CT (Gersib 2023). In individuals with MID‐BIF, this may be even more of a challenge because it is possible that CT in individuals with MID‐BIF differs from that of nondisabled individuals. This is to be expected due to their limited vocabulary; many individuals with MID‐BIF experience difficulties in communication skills and in expressing their feelings and thoughts (American Psychiatric Association 2022). This may impact the way CT is expressed. As far as we know, CT has not been studied before in individuals with MID‐BIF and SUD. Knowledge about CT in individuals with MID‐BIF and SUD is important for therapists to also recognise CT that is not so explicit or hidden. In addition, knowledge about the sequential association between therapist and client language in SUD treatment may provide clinical implications to further improve the implementation of MI in individuals with MID‐BIF and SUD.

The aim of this study was to examine the sequential association between therapist MI skills and client language during SUD treatment in individuals with MID‐BIF. In this study, participants had followed Take it Personal!+, which is a personality‐targeted MI‐CBT treatment programme for individuals with MID‐BIF and SUD aiming to reduce SU (Gosens et al. 2021). We hypothesise the same sequential associations as found in people without MID‐BIF: MI‐consistent language, questions of CT and reflections of CT will be followed by CT. MI‐inconsistent language, questions of CCT and reflections of CCT will be followed by CCT in participants with MID‐BIF during Take it Personal!+. In addition, we will explore how participants express CT by identifying the different utterances of CT, which will be illustrated by participants' citations.

## 2 Material and Methods

### 2.1 Study Design and Participants

There were four participants and their therapist, who also participated in an effectiveness study of the personality‐targeted MI‐CBT treatment programme Take it Personal!+ (see Gosens et al. 2024). Participants in this study were in specialised MID‐BIF care, and classification has been done based on a comprehensive clinical assessment of intellectual impairments and deficits in adaptive functioning conducted by a multidisciplinary team. This assessment is typically supported by standardised intelligence testing, and, consistent with DSM‐5 criteria, the impairments were established to have been present during the developmental period. The current study was approved by the Ethics Committee Social Sciences of the Radboud University (ECSW‐2020‐080). Sessions were audio recorded between summer 2020 and summer 2021. Clients and their therapists provided written informed consent for audio recording. The Take it Personal!+ treatment programme consists of 22 sessions, and in the first sessions the focus is on MI (Gosens et al. 2021). For the current study, the first 6–12 treatment sessions of Take it Personal!+ were audio recorded to examine the sequential relationship between therapist and client language. Data were based on the first 12 sessions of Participant 1. Participant 2 received only the first six sessions of the treatment because she then decided she needed a clinical SUD treatment. Of Participant 3, the audio recording of Session 12 was lacking because the therapist forgot to record the session. Of Participant 4 we were not able to transcribe and code the last six sessions because of financial constraints. All participants were in MID‐BIF care, and the characteristics of the participants are shown in Table 1. Three therapists carried out the treatment. All were experienced in providing treatment to individuals with MID‐BIF and were trained in the Take it Personal!+ treatment protocol (Gosens et al. 2021). Two therapists were trained in MI by the second author, a member of the MI Network of Trainers and all therapists followed a certified e‐learning in MI. In some of the sessions the confidant of the participant (i.e., someone from the social network or professional caregivers) was present according to the treatment protocol. However, confidant language was not analysed because it is outside the scope of this study.

**TABLE 1:** Participants' characteristics.

	Age	Sex	IQ	SUD	Treatment goal (target behaviour)	Recorded sessions (*n*)
P1	35	M	Index scores between 66 and 86^a^	Moderate alcohol	Maximum three glasses a day	12
P2	18	F	TIQ: 80	Severe cannabis	Quit cannabis	6
P3	23	M	TIQ: 78	Severe cannabis	Decrease cannabis	11
P4	23	F	Index scores between 60 and 79^a^	Severe cannabis	Quit cannabis	6

Abbreviations: F, female; IQ, intelligence quotient; M, male; SUD, substance use disorder based on DSM‐5 criteria; TIQ, total IQ.

^a^ Disharmonic profile, index scores of verbal comprehension index, perceptual reasoning index, working memory index and processing speed index.

### 2.2 Coding Process

First, audio recordings of treatment sessions were transcribed verbatim. Second, the first author parsed transcripts into therapist and client utterances by listening to the audio recordings using the Motivational Interviewing Sequential Code for Observing Process Exchanges (MI‐SCOPE) (Martin et al. 2005). The MI‐SCOPE consists of 34 therapist behaviour codes (e.g., simple reflections, affirm, general information, support, direct, open double‐sided question) and 17 client behaviour codes (e.g., follow neutral, ask, ability to change, ability counter to change, desire to change, desire counter to change). Third, trained bachelor's and master's students listened to the audio recordings to assess the global ratings based on the Manual for the Motivational Interviewing Skill Code version 2.1 (MISC 2.1) and to provide an overall impression of the therapists' performance (Miller et al. 2008). These global ratings consist of three dimensions, that is, acceptance, empathy and MI spirit, which can be scored on a 7‐point Likert scale (i.e., ‘1’ = *low* to ‘7’ = *high*). In this study, the therapists' performance can be marked as good and in line with another study (Dobber et al. 2020), as the therapists had a mean acceptance score of 4.5 (SD = 0.76), a mean empathy score of 4.5 (SD = 0.62) and a mean MI‐spirit score of 4.9 (SD = 0.60) over 33 sessions (i.e., global ratings of two sessions were missing). Fourth, the bachelor's and master's students coded the transcripts by listening to the audio recordings and using the MI‐SCOPE. The first author and two coders were trained in using the MI‐SCOPE and the MISC 2.1 for global ratings in a 5‐day course by the second author and Jos Dobber, and the first author trained another three coders. Before data collection started, the agreement between the trainer's transcript and the coder's transcripts was calculated and reached a conformity of 87%. During data collection, coding dilemmas were discussed in a biweekly coder–trainer meeting.

In line with other studies and in accordance with MI theory (Barnett et al. 2014; Dobber et al. 2021; Gaume et al. 2008; Lindqvist et al. 2017; Moyers et al. 2009), the therapist and client behaviour MI‐SCOPE codes were collapsed into different categories to conduct analyses. See Table 2 for the collapsed therapist codes into the categories MI‐consistent, MI‐inconsistent, General information‐MI and other (Table 2). Some studies also included reflections and questions in the MI‐consistent category as also described in the MISC 2.1 (Gaume et al. 2008; Miller et al. 2008). We decided not to include questions and reflections in order to separately analyse the effects of this language, resulting in the following categories: questions of CT (i.e., open and closed questions), questions of CCT (i.e., open and closed questions), two‐sided questions (i.e., open and closed questions about CT and CCT), neutral questions (i.e., open and closed questions), reflections of CT (i.e., simple and complex), reflections of CCT (i.e., simple and complex), two‐sided reflections (i.e., simple and complex) and neutral reflections (i.e., simple and complex). Client behaviour codes were collapsed into three categories (Table 2). Intra‐rater agreement (i.e., coded by the same coder) was calculated over 10% of the audio‐recorded sessions (*n* = 4) (kappa of collapsed MI‐codes = 0.81) and inter‐rater agreement (i.e., between coders) over 23% of sessions (*n* = 8) (kappa of collapsed MI‐codes = 0.88). Kappa scores can be marked as excellent (Cohen 1960).

**TABLE 2:** Collapsed categories of MI‐SCOPE codes.

Therapist behavioural codes	Client behavioural codes
MI‐consistent	Change talk (CT)
Affirm	Commitment
Emphasise control	Desire
Permission seeking	Ability
Support	Need
Advise with permission	Reasons
	Taking steps
	Other CT
MI‐inconsistent	Counter change talk (CCT)
Advise without permission	Commitment counter
Confront	Desire counter
Direct	Ability counter
Opinion	Need counter
Warn	Reasons counter
	Taking steps counter
	Other CCT
General information‐MI	Neutral (NEU)
General information	Ask
Feedback	Follow/neutral
	Not encodable
Other	
Facilitate	
Filler	
Self‐disclosure	
Raise concern	
Structure	
Not encodable	

Abbreviation: MI, motivational interviewing.

### 2.3 Data Analysis

#### 2.3.1 Sequential Relationships Between Therapist and Client Language

Transitional probabilities were calculated using Generalized Sequential Querier (GSEQ 5.1) software (Bakeman and Quera 2016). GSEQ allows for the analyses of sequential observational data. Transitional probability measures the probability that therapist language was immediately followed by client language (e.g., the chance that a reflection of CT is immediately followed by CT). We calculated observed and expected frequencies of the transitions, transitional probabilities, odds ratio (OR) for the transitions and 95% confidence interval (CI) in GSEQ 5.1 with therapist behaviour codes as given and client behaviour codes as target, pooled over the 4 clients and 35 sessions. In computing reliable transitional probabilities, the expected frequency should be minimally three, although a minimum of five is preferable (Martin et al. 2005). We excluded the four transitions with an expected frequency of five or less from analysis.

#### 2.3.2 CT in Individuals With MID‐BIF and SUD

To describe the CT of participants with MID‐BIF and SUD during Take it Personal!+, we calculated frequencies of the MI‐SCOPE codes of CT in IBM SPSS Statistics 29 over sessions and clients. Four missing codes were excluded from the analysis. In addition, examples of different MI‐SCOPE codes of CT were illustrated with citations derived from the transcripts.

## 3 Results

### 3.1 Transitions From Therapist Language to Client Language During Take It Personal!+

Table 3 shows results of the sequential analyses of transitions from therapist to client language. Results show, among others, that questions and reflections directed at SU change (questions‐CT and reflections‐CT) were the main therapist's MI skills that resulted in CT by clients. Surprisingly, therapist language in the MI‐consistent category was not more likely to be followed by CT. CCT was mainly elicited by questions and reflections directed at not changing SU (questions‐CCT and reflections‐CCT). Also, therapist language in the MI‐inconsistent category was not more likely to be followed by CCT. Utterances in the general information‐MI category and the other category were more likely to be followed by neutral client language.

**TABLE 3:** Transitions from therapist language to client language.

Transitions	Observed frequency	Expected frequency	Transitional probability	Odds ratio	Confidence limits
MI‐consistent → CT	44	43	0.11	1.02	0.74–1.40
MI‐consistent → CCT	13	25	0.03	0.49^a^	0.28–0.86
MI‐consistent → NEU	355	344	0.86	1.25	0.94–1.66
MI‐inconsistent → CT	8	11	0.08	0.74	0.36–1.52
MI‐inconsistent → CCT	6	6	0.06	0.99	0.43–2.26
MI‐inconsistent → NEU	86	83	0.86	1.22	0.69–2.16
Questions					
Question‐CT → CT	186	37	0.52	10.89^a^	8.67–13.54
Question‐CT → CCT	18	22	0.05	0.82^a^	0.51–1.33
Question‐CT → NEU	150	295	0.43	0.13^a^	0.11–0.17
Question‐CCT → CT	42	29	0.15	1.54^a^	1.10–2.15
Question‐CCT → CCT	129	17	0.46	16.20^a^	12.61–20.80
Question‐CCT → NEU	106	231	0.38	0.11^a^	0.09–0.15
Two‐sided question → CT	80	27	0.31	4.04^a^	3.08–5.29
Two‐sided question → CCT	40	16	0.16	2.96^a^	2.09–4.18
Two‐sided question → NEU	137	214	0.52	0.22^a^	0.17–0.28
Question neutral → CT	79	171	0.05	0.40^a^	0.31–0.50
Question neutral → CCT	43	99	0.03	0.38^a^	0.28–0.52
Question neutral → NEU	1502	1354	0.92	2.71^a^	2.24–3.28
Reflections					
Reflection‐CT → CT	223	63	0.37	5.92^a^	4.96–7.07
Reflection‐CT → CCT	15	36	0.02	0.38^a^	0.23–0.65
Reflection‐CT → NEU	361	499	0.61	0.28^a^	0.23–0.33
Reflection‐CCT → CT	29	43	0.07	0.64^a^	0.44–0.94
Reflection‐CCT → CCT	125	25	0.31	8.02^a^	6.40–10.06
Reflection‐CCT → NEU	255	341	0.62	0.31^a^	0.25–0.38
Two‐sided reflection → NEU	33	36	0.74	0.66	0.32–1.33
Reflection neutral → CT	24	69	0.04	0.31^a^	0.21–0.47
Reflection neutral → CCT	14	40	0.02	0.32^a^	0.19–0.55
Reflection neutral → NEU	617	546	0.94	3.38^a^	2.43–4.71
General information‐MI → CT	153	339	0.05	0.34^a^	0.29–0.41
General information‐MI → CCT	86	196	0.03	0.35^a^	0.28–0.43
General information‐MI → NEU	2984	2688	0.93	3.12^a^	2.71–3.60
Other → CT	375	412	0.10	0.86^a^	0.75–0.97
Other → CCT	228	238	0.06	0.94	0.80–1.10
Other → NEU	3314	3267	0.85	1.14^a^	1.03–1.27

Abbreviations: CCT, counter change talk; CT, change talk; MI, motivational interviewing; NEU, neutral.

^a^ Transition is significant.

### 3.2 CT in Individuals With MID‐BIF and SUD During Take It Personal!+

Table 4 provides an overview of frequencies of CT utterances pooled over 4 participants and 35 sessions. ‘Reasons’ was the most frequently used CT, which are utterances reflecting reasons to change SU. The second most common was ‘other CT’, which are utterances of CT that cannot be coded as one of the other CT codes. The third most commonly used CT pertains to ‘ability’, which refers to utterances about the ability to change SU. Fourth, ‘taking steps’ are utterances about recent steps that are taken regarding changing SU, and fifth, ‘desire’: utterances about the desire to change SU. Then there is ‘need’, referring to utterances about the need to change SU and finally—the least frequently used CT—there was ‘commitment’, which refers to utterances about the commitment to change SU. Citations of the different CT are also provided in Table 4, showing participants were able to express CT in their own words (e.g., ‘June first I want to get rid of it’) and sometimes the therapist provided words to the desire of the client (e.g., ‘So with that you say I know what I want, I want to stop completely. I want to stop smoking weed’.), which was answered positively (i.e., ‘yes’).

**TABLE 4:** Frequencies and citations of client CT utterances.

CT category, *N* (%)	Citation
Commitment, 31 (2.1%)	P3: I will keep going [treatment] until I stop smoking weed
	P4: June first I want to get rid of it [smoking weed]
Desire, 123 (8.2%)	Therapist: So with that you say I know what I want, I want to stop completely. I want to stop smoking weed. P2: Yes
	P4: Hm and then if I cannot sleep,^a^ then I have more desire to start smoking joints again and that is something I do not want any more
Ability, 293 (19.7%)	P2: I just need help with that [stop cannabis use] and that is just it
	P3: Yes I really have that feeling of yes I will get there [decreasing cannabis use] I did not have that feeling at all at the beginning of this process [treatment] but now more and more
Need, 46 (3.1%)	P2: But that is not my problem [denying addiction] either I guess. I just know that I really need to get rid of it [addiction]
	P3: No, I am now really aware of that I need to work on it [decreasing cannabis use]
Reasons, 425 (28.5%)	P1: Yes that I almost yes almost literally saw someone die [because of drinking]. Yes like that can also be a final station for me, rather not yes
	P3: My mother that is also what makes me want to stop smoking weed. Especially, because yes I have been asking her a lot of money lately just for smoking weed and for smoking and further actually for other things quite little
Taking steps, 220 (14.8%)	P1: They did have beer, but I said do something else [drink without alcohol]
	P4: Because now, when you came, I did not smoke weed on beforehand either. So I do can do it
Other CT, 353 (23.7%)	P2: Postpone longer. Not immediately if I feel like smoking weed, or I want to or, yes not to do that right away. To postpone that
	P4: I do not like to walk around at home or at work stoned in the morning. I do not like that anymore

^a^ To understand the citation the whole citation was provided while actually the first part of the sentence is ‘reason’ and only the second part is ‘desire’.

## 4 Discussion

The first aim of this study was to examine sequential associations between therapist and client language during SUD treatment in individuals with MID‐BIF. Consistent with our hypothesis, the results showed that questions of CT and reflections of CT were likely to be followed by CT, indicating both skills are powerful in evoking CT. These outcomes are also in line with MI theory (Miller and Rollnick 2023) and earlier studies in individuals without MID‐BIF (Barnett et al. 2014; Berman et al. 2019; Lindqvist et al. 2017). In addition, questions of CCT and reflections of CCT were followed by CCT. This is also in line with MI theory (Miller and Rollnick 2023) and earlier studies in individuals without MID‐BIF (Barnett et al. 2014; Berman et al. 2019; Lindqvist et al. 2017). Inconsistent with our hypothesis, the results showed no sequential associations between the MI‐consistent category and CT, or between MI‐inconsistent category and CCT. The second aim was to identify the different utterances of CT in individuals with MID‐BIF during Take it Personal!+. Results showed that all categories of CT, as studied in individuals without MID‐BIF, were also expressed by individuals with MID‐BIF during SUD treatment.

We found no support for the hypothesised sequential relationship between the MI‐consistent category and CT. As mentioned earlier, we did not include questions and reflections in the MI‐consistent category, as some other studies did, and as shown, this can lead to CT. In line with our study, Gaume et al. (2010) also found no sequential association between the MI‐consistent category and CT after removing questions and reflections of this category. However, the study of Lindqvist et al. (2017) used the same MI‐consistent category as we did and did show the MI‐consistent category was more likely to be followed by CT. A possible explanation for this difference may be found in the characteristics of participants. Individuals with MID‐BIF might need more direct language to respond directly with CT, as questions and reflections directly target CT (e.g., ‘So you want to stop smoking weed’) and other MI‐consistent therapist skills, such as affirming (e.g., ‘Very good of you that you reduced your substance use’), do not. In addition, the target group mostly has a slower processing speed. It is possible that languages of the MI‐consistent category eventually will lead to CT in individuals with MID‐BIF; albeit, in line with other scholars, we only analysed direct response (i.e., lag 1). However, our study did show a sequential association of the MI‐consistent category, as it was less likely to be followed by CCT, which is in line with the Lindqvist et al. (2017) study.

Also, the MI‐inconsistent category was not followed by CCT, which we expected based on MI theory and earlier studies (Gaume et al. 2010; Magill et al. 2018). Some other studies also did not find support for this hypothesis (Gaume et al. 2008; Lindqvist et al. 2017). Moreover, in our study, MI‐inconsistent language was also not more or less likely to be followed by CT or NEU. It may be concluded that MI‐inconsistent language had no effect on direct (i.e., lag 1) client language. Possible explanations may also be regarding the direct language and slower processing speed.

The results also showed some remarkable findings. Questions of CCT were more likely to be followed by CCT, which is in line with MI theory. However, questions of CCT were also more likely to be followed by CT. This sequential relationship is not expected based on the MI theory and an explanation for this relationship remains to be explored. Further research is necessary. In addition, two‐sided questions were more likely to be followed by CT and CCT. This can be explained by the fact that a two‐sided question includes a question about CT and CCT (e.g., ‘Can you tell me the pros and cons of SU?’). Which explains the fact that a client will react either with CT or CCT.

The second aim of this study was to identify the different utterances of CT in individuals with MID‐BIF during SUD treatment. Results showed that participants expressed all utterances of CT. The citations indicated participants were able to express CT in their own words, and sometimes they needed the therapist to express their desire, which they confirmed as CT. Thereby it should be noted that therapists sometimes need to provide words to the desire of the client since individuals with MID‐BIF may experience difficulties in expressing their feelings and thoughts. ‘Reasons’ was the most frequently expressed CT in this study, and ‘commitment’ was the least frequently expressed CT. We only analysed the first 6–12 sessions. It may be possible that commitment will increase in the later sessions, as according to the self‐determination theory, motivation changes from more external (e.g., reasons and need) to more internal (commitment). The associations between CT utterances and SU outcome were studied in individuals without MID‐BIF, showing associations between reasons utterances and reductions in SU (Baer et al. 2008; Osilla et al. 2015) and also one study with commitment utterances (Osilla et al. 2015). Which CT utterances are associated with SU outcome in individuals with MID‐BIF is still unknown and needs to be further explored.

Studies in individuals without MID‐BIF showed associations between CT and a decrease of SU (Houck et al. 2018; Vader et al. 2010). If this causal relationship is the same for individuals with MID‐BIF, the results of this study support the MI theory of implementing reflections of CT and questions of CT in the target group to evoke CT, which probably leads to a decrease in SU. Besides the MI skills, an MI therapist also utilises the MI attitude (Miller and Rollnick 2023). An earlier study in individuals without MID‐BIF showed the relational component also influences client language and SU outcome (Gaume et al. 2021). In further research testing if CT is related to a decrease of SU in individuals with MID‐BIF and SUD, it is recommended to not only include the MI skills but also the relational component.

In some of the included sessions, a confidant of the client (i.e., someone from the social network or professional caregivers) was also present according to the treatment protocol. In this study, the confidant's language was not studied because it was out of the scope. However, client language can be influenced by confidant language. Further analysis of the interaction between client, confidant and therapist language is needed to determine the impact of the confidant. As in clinical practice, a caregiver is often present during treatment; the presence of a caregiver in SUD treatment in individuals with MID‐BIF seems to be supportive (Gosens et al. 2025).

This study has some limitations. First, the generalisability of the results is limited. Only four participants were included in this study. Although many sessions per individual were studied (25 sessions of approximately 45–60 min were coded), further research on a larger scale is necessary to increase generalisability. Second, only the direct (i.e., lag 1) sequential relationship was studied, and it is possible that clients react later to therapist language. Third, this study only focused on therapists' MI skills and the subsequent response of individuals with MID‐BIF, and although it is hypothesised that CT is related to a decrease in SU, this was not tested in this study. Further research is necessary to test this association.

Concluding, the present study contributes to the understanding of MI and CT in individuals with MID‐BIF and SUD. Showing support for sequential relationships between therapist and client language in individuals with MID‐BIF and SUD as set in MI theory and in line with studies in individuals without MID‐BIF. Indicating questions of CT and reflections of CT as powerful skills in evoking CT in individuals with MID‐BIF during SUD treatment. Furthermore, this study showed individuals with MID‐BIF are able to express CT with their own words and sometimes need the therapist to provide words for their desire. Further research on a larger scale is necessary to test if CT is also related to a decrease of SU in individuals with MID‐BIF and SUD.

## Author Contributions

**Lotte C. F. Gosens:** conceptualisation, methodology, data collection, formal analysis, writing – original draft. **Jannet M. de Jonge:** conceptualisation, methodology, data collection, formal analysis, writing – review and editing, supervision. **Robert Didden:** conceptualisation, writing – review and editing, supervision. **Roy Otten:** conceptualisation, writing – review and editing, supervision. **Evelien A. P. Poelen:** conceptualisation, methodology, data collection, formal analysis, writing – review and editing, supervision, funding acquisition.

## Funding

This study was funded by the Netherlands Organisation for Health Research and Development (ZonMw project 636330005).

## Conflicts of Interest

The authors declare no conflicts of interest.

## Data Availability

Research data are not shared.

## References

[jar70169-bib-0001] American Psychiatric Association . 2022. Diagnostic and Statistical Manual of Mental Disorders. 5th ed. American Psychiatric Association.

[jar70169-bib-0002] Baer, J. S. , B. Beadnell , S. B. Garret , B. Hartzler , E. A. Wells , and P. L. Peterson . 2008. “Adolescent Change Language Within a Brief Motivational Intervention and Substance Use Outcomes.” Psychology of Adddictive Behaviors 22, no. 4: 570–575. 10.1037/a0013022. PMC2605642 19071983

[jar70169-bib-0003] Bakeman, R. , and V. Quera . 2016. “Generalized Sequental Querier (GSEQ): A Program for Analyzing Sequential Data.” Version 5.1.23.

[jar70169-bib-0004] Barnett, E. , D. Spruijt‐Metz , T. B. Moyers , et al. 2014. “Bidirectional Relationships Between Client and Counselor Speech: The Importance of Reframing.” Psychology of Addictive Behaviors 28, no. 4: 1212–1219. 10.1037/a0036227. 24955660 PMC4274216

[jar70169-bib-0005] Berman, A. H. , H. Lindqvist , H. Källmén , N. Durbeej , U. Hermansson , and L. Forsberg . 2019. “Counselor and Drug Detox Inpatient Verbal Behaviors in a Single Session of Motivational Interviewing and Subsequent Substance Use‐Related Patient Outcomes.” International Journal of Mental Health and Addiction 17, no. 1: 73–88. 10.1007/s11469-017-9866-4.

[jar70169-bib-0006] Cohen, J. A. 1960. “Coefficient of Agreement for Nominal Scales.” Educational and Psychological Measurement 20: 37–46. 10.1177/001316446002000104.

[jar70169-bib-0008] Didden, R. , J. VanDerNagel , M. Delforterie , and N. van Duijvenbode . 2020. “Substance Use Disorders in People With Intellectual Disability.” Current Opinion in Psychiatry 33, no. 2: 124–129. 10.1097/YCO.0000000000000569. 31743126

[jar70169-bib-0009] Dobber, J. , C. Latour , B. Van Meijel , et al. 2020. “Active Ingredients and Mechanisms of Change in Motivational Interviewing for Medication Adherence. A Mixed Methods Study of Patient‐Therapist Interaction in Patients With Schizophrenia.” Frontiers in Psychiatry 11: 78. 10.3389/fpsyt.2020.00078. 32265746 PMC7105777

[jar70169-bib-0010] Dobber, J. , M. Snaterse , C. Latour , et al. 2021. “Active Ingredients and Mechanisms of Change in Motivational Interviewing for Smoking Cessation in Patients With Coronary Artery Disease: A Mixed Methods Study.” Frontiers in Psychology 12: 3389. 10.3389/fpsyg.2021.599203. PMC8258345 34239470

[jar70169-bib-0011] Frielink, N. , C. Schuengel , A. A. C. Kroon , and P. J. C. M. Embregts . 2015. “Pretreatment for Substance‐Abusing People With Intellecutal Disabilites: Intervening on Autonomous Motivation for Treatment Entry.” Journal of Intellectual Disability Research 59: 1168–1182. 10.1111/jir.12221. 26369922

[jar70169-bib-0012] Gaume, J. , N. Bertholet , M. Faouzi , G. Gmel , and J. B. Daeppen . 2010. “Counselor Motivational Interviewing Skills and Young Adult Change Talk Articulation During Brief Motivational Interventions.” Journal of Substance Abuse Treatment 39: 272–281. 10.1016/j.jsat.2010.06.010. 20708900

[jar70169-bib-0013] Gaume, J. , G. Gmel , M. Faouzi , and J. B. Daeppen . 2008. “Counsellor Behaviours and Patient Language During Brief Motivational Interventions: A Sequential Analysis of Speech.” Addiction 103: 1793–1800. 10.1111/j.1360-0443.2008.02337.x. 19032529

[jar70169-bib-0014] Gaume, J. , M. Magill , G. Gmel , and J. B. Daeppen . 2021. “Motivational Interviewing Technical and Relational Skills, Change Talk, and Alcohol Outcomes—A Moderated Mediation Analysis.” Journal of Consulting and Clinical Psychology 89, no. 8: 707–716. 10.1037/ccp0000666. 34472897

[jar70169-bib-0015] Gersib, J. A. 2023. “Supporting Middle School Student Behavior Change Through Motivational Interviewing.” Beyond Behavior 32, no. 2: 115–127. 10.1177/10742956221122723.

[jar70169-bib-0016] Gosens, L. C. F. , R. Didden , J. M. de Jonge , et al. 2025. “Experiences With Take It Personal!+ From People With Mild Intellectual Disability or Borderline Intellectual Functioning and Substance Use Disorder and Their Confidants: A Qualitative Study.” Journal of Mental Health Research in Intellectual Disabilities 18: 221–238. 40201031 10.1080/19315864.2025.2459418 PMC11974916

[jar70169-bib-0017] Gosens, L. C. F. , R. Otten , J. M. de Jonge , et al. 2021. “Development of a Personalized Substance Use Disorder Treatment for People With Mild Intellectual Disabilities or Borderline Intellectual Functioning: An Intervention Mapping Approach.” Journal of Intellectual & Developmental Disability 47: 131–140. 10.3109/13668250.2021.1925529. 39818578

[jar70169-bib-0018] Gosens, L. C. F. , E. A. P. Poelen , R. Didden , et al. 2024. “Evaluating the Effectiveness of Take It Personal!+ In People With Mild Intellectual Disability or Borderline Intellectual Functioning and Substance Use Disorder: A Multiple Baseline Single‐Case Experimental Study.” Behavior Therapy 55, no. 2: 331–346. 10.1016/j.beth.2023.07.007. 38418044

[jar70169-bib-0019] Houck, J. M. , J. K. Manuel , and T. B. Moyers . 2018. “Short‐ and Long‐Term Effects of Within‐Session Client Speech on Drinking Outcomes in the COMBINE Study.” Journal of Studies on Alcohol and Drugs 79, no. 2: 217–222. 10.15288/jsad.2018.79.217. 29553348 PMC6019773

[jar70169-bib-0020] Kouimtsidis, C. , A. Bosco , K. Scior , et al. 2017. “A Feasibility Randomised Controlled Trial of Extended Brief Intervention for Alcohol Misuse in Adults With Mild to Moderate Intellectual Disabilities Living in the Community; the EBI‐LD Study.” Trials 18: 216. 10.1186/s13063-017-1953-0. 28499440 PMC5427589

[jar70169-bib-0021] Lindqvist, H. , L. Forsberg , P. Enebrink , G. Andersson , and J. Rosendahl . 2017. “The Relationship Between Counselors' Technical Skills, Clients' In‐Session Verbal Responses, and Outcome in Smoking Cessation Treatment.” Journal of Substance Abuse Treatment 77: 141–149. 10.1016/j.jsat.2017.02.004. 28245946

[jar70169-bib-0022] Magill, M. , T. R. Apodaca , B. Borsari , et al. 2018. “A Meta‐Analysis of Motivational Interviewing Process: Technical, Relational, and Conditional Process Models of Change.” Journal of Consulting and Clinical Psychology 86, no. 2: 140–157. 10.1037/ccp0000250. 29265832 PMC5958907

[jar70169-bib-0023] Magill, M. , J. Gaume , T. R. Apodaca , et al. 2014. “The Technical Hypothesis of Motivational Interviewing: A Meta‐Analysis of MI's Key Causal Model.” Journal of Consulting and Clinical Psychology 82, no. 6: 973–983. 10.1037/a0036833. 24841862 PMC4237697

[jar70169-bib-0024] Martin, T. , T. B. Moyers , J. Houck , P. Christopher , and W. R. Miller . 2005. Motivational Interviewing Sequential Code for Observing Process Exchanges (MI‐SCOPE). Coder's Manual. University of New Mexico. https://casaa.unm.edu/download/scope.pdf.

[jar70169-bib-0025] Mendel, E. , and J. Hipkins . 2002. “Motivating Learning Disabled Offenders With Alcohol‐Related Problems: A Pilot Study.” British Journal of Learning Disabilities 30, no. 4: 153–158. 10.1046/j.1468-3156.2002.00209.x.

[jar70169-bib-0026] Miller, W. R. , T. B. Moyers , D. Ernst , and P. Amhrein . 2008. Manual for the Motivational Interviewing Skill Code (MISC) Version 2.1. University of New Mexico.

[jar70169-bib-0027] Miller, W. R. , and S. Rollnick . 2023. Motivational Interviewing: Helping People Change and Grow. 4th ed. Guilford Press.

[jar70169-bib-0028] Moyers, T. B. , T. Martin , J. M. Houck , P. J. Christopher , and J. S. Tonigan . 2009. “From In‐Session Behaviors to Drinking Outcomes: A Causal Chain for Motivational Interviewing.” Jouranl of Consulting and Clinical Psychology 77, no. 6: 1113–1124. https://10.1037/a0017189. 10.1037/a0017189 PMC2819223 19968387

[jar70169-bib-0029] Osilla, K. C. , J. A. Ortiz , J. N. V. Milles , E. R. Pedersen , J. M. Houck , and E. J. D'Amico . 2015. “How Group Factors Affect Adolescent Change Talk and Substance Use Outcomes: Implications for Motivational Interviewing Training.” Journal of Counseling Psychology 62, no. 1: 79–86. 10.1037/cou0000049. 25602608 PMC4300532

[jar70169-bib-0030] Riper, H. , G. Andersson , S. B. Hunter , J. de Wit , M. Berking , and P. Cuijpers . 2014. “Treatment of Comorbid Alcohol Use Disorders and Depression With Cognitive Behavioural Therapy and Motivational Interviewing: A Meta‐Analysis.” Addiction 109, no. 3: 394–406. 10.1111/add.12441. 24304463 PMC4227588

[jar70169-bib-0031] Rodriguez, M. , S. T. Walters , J. M. O. Houck , J. A. Ortiz , and F. S. Taxman . 2017. “The Language of Change Among Criminal Justice Clients: Counselor Language, Client Language, and Client Substance Use Outcomes.” Journal of Clinical Psychology 74: 626–636. 10.1002/jclp.22534. 28940435 PMC5862754

[jar70169-bib-0032] Smedslund, G. , R. C. Berg , K. T. Hammerstrøm , et al. 2011. “Motivational Interviewing for Substance Abuse.” Campbell Systematic Reviews 7, no. 1: 1–126. 10.4073/csr.2011.6. PMC8939890 21563163

[jar70169-bib-0033] Vader, A. M. , S. T. Walters , G. C. Prabhu , J. M. Houck , and C. A. Field . 2010. “The Language of Motivational Interviewing and Feedback: Counselor Language, Client Language, and Client Drinking Outcomes.” Psychology of Addictive Behaviors 24, no. 2: 190–197. 10.1037/a0018749. 20565145 PMC2891547

[jar70169-bib-0034] van Duijvenbode, N. , and J. E. L. VanDerNagel . 2019. “A Systematic Review of Substance Use (Disorder) in Individuals With Mild to Borderline Intellectual Disability.” European Addiction Research 25, no. 6: 263–282. 10.1159/000501679. 31330514 PMC6888885

[jar70169-bib-0035] van Duijvenbode, N. , J. E. L. VanDerNagel , R. Didden , et al. 2015. “Substance Use Disorders in Individuals With Mild to Borderline Intellectual Disability: Current Status and Future Directions.” Research in Developmental Disabilities 38: 319–328. 10.1016/j.ridd.2014.12.029. 25577182

